# Gyrification in relation to cortical thickness in the congenitally blind

**DOI:** 10.3389/fnins.2022.970878

**Published:** 2022-11-09

**Authors:** Isabel Arend, Kenneth Yuen, Or Yizhar, Daniel-Robert Chebat, Amir Amedi

**Affiliations:** ^1^The Joseph Sagol Neuroscience Center, The Chaim Sheba Medical Center, Ramat Gan, Israel; ^2^Neuroimaging Center (NIC), Focus Program Translational Neuroscience, Johannes Gutenberg University Medical Center, Mainz, Germany; ^3^Leibniz Institute for Resilience Research, Mainz, Germany; ^4^The Institute for Brain, Mind and Technology, The Baruch Ivcher School of Psychology, Reichman University, Herzliya, Israel; ^5^Max Planck Institute for Human Development, Research Group Adaptive Memory and Decision Making, Berlin, Germany; ^6^Max Planck Institute for Human Development, Berlin, Germany; ^7^Department of Psychology, Navigation and Accessibility Research Center of Ariel University (NARCA), Ariel, Israel; ^8^Visual and Cognitive Neuroscience Laboratory (VCN Lab), Department of Psychology, Faculty of Social Sciences and Humanities, Ariel University, Ariel, Israel

**Keywords:** vision, voxel-based morphometry, MRI, cortical thickness, gyrification, congenital blindness, late onset blindness, cross-modal plasticity

## Abstract

Greater cortical gyrification (GY) is linked with enhanced cognitive abilities and is also negatively related to cortical thickness (CT). Individuals who are congenitally blind (CB) exhibits remarkable functional brain plasticity which enables them to perform certain non-visual and cognitive tasks with supranormal abilities. For instance, extensive training using touch and audition enables CB people to develop impressive skills and there is evidence linking these skills to cross-modal activations of primary visual areas. There is a cascade of anatomical, morphometric and functional-connectivity changes in non-visual structures, volumetric reductions in several components of the visual system, and CT is also increased in CB. No study to date has explored GY changes in this population, and no study has explored how variations in CT are related to GY changes in CB. T1-weighted 3D structural magnetic resonance imaging scans were acquired to examine the effects of congenital visual deprivation in cortical structures in a healthy sample of 11 CB individuals (6 male) and 16 age-matched sighted controls (SC) (10 male). In this report, we show for the first time an increase in GY in several brain areas of CB individuals compared to SC, and a negative relationship between GY and CT in the CB brain in several different cortical areas. We discuss the implications of our findings and the contributions of developmental factors and synaptogenesis to the relationship between CT and GY in CB individuals compared to SC. F.

## Introduction

The congenitally blind (CB) brain exhibits remarkable functional plasticity (Bavelier and Neville, 2002; Merabet and Pascual-Leone, 2010; Voss, 2013), influencing both white and gray matter (Noppeney et al., 2005; Shimony et al., 2006; Ptito et al., 2008; Jiang et al., 2009; Modi et al., 2012; Reislev et al., 2016, 2017). Indeed, there is a cascade of anatomical (Noppeney et al., 2005; Yang et al., 2014; Cecchetti et al., 2016), structural (Shimony et al., 2006; Bridge et al., 2009; Bridge and Watkins, 2019), morphological (Park et al., 2009), morphometric (Rombaux et al., 2010; Tomaiuolo et al., 2014; Aguirre et al., 2016; Maller et al., 2016), functional-connectivity (Heine et al., 2015), and metabolic changes (de Volder et al., 1997) in visual areas. Complete absence of vision from birth alters the cortical thickness (CT) of the brain and specifically in the primary visual cortex, which has been shown to be increased compared to sighted individuals (Jiang et al., 2009; Park et al., 2009; Kupers and Ptito, 2014; Anurova et al., 2015). CT is defined as the distance between the white/gray matter surface and pial surface. There is increased thickness in cortical visual areas of the CB relative to SC including the left visual association cortex (Jiang et al., 2009), the pericalcarine sulcus, lingual gyrus, right rostral middle frontal gyrus, left caudate and anterior cingulate cortices (Park et al., 2009). Both CB and LB showed thinner entorhinal cortex relative to SC (Jiang et al., 2009). These brain modifications are believed to be triggered at first by sensory deprivation (i.e., disuse related mechanisms), and later by the training of the other senses (i.e., training induced brain plasticity) (Chebat et al., 2020). The changes in CT of the CB brain have been explained in terms of disuse related mechanisms, but also in terms of cross-modal functional recruitment of brain areas (Jiang et al., 2009; Park et al., 2009; Voss and Zatorre, 2012; Anurova et al., 2015).

During the early stages of brain development there is notable increase in the formation of synaptic contacts, immediately followed by sensory dependent pruning of inactive synapses (Inuggi et al., 2020). In the visual cortex, synaptogenesis reaches a maximum of synaptic density around 8 months to 1 year of age. A long period of pruning, which lasts up to 11 years of age, eliminates about 40% of synapses. Prolonged visual deprivation during development alters pruning mechanisms which are dependent on sensory input (Pascual-Leone et al., 2005). Increased CT in the visual cortex in CB has been attributed to disuse related mechanisms during development (Jiang et al., 2009). In line with this time frame, changes in the thickness of the cortex are not observed in late blind (LB) individuals (Jiang et al., 2009; Park et al., 2009; Voss and Zatorre, 2012; Anurova et al., 2015), and CT gradually is linked with the age of blindness onset (Li et al., 2017). These differences between CB and LB individuals suggest that CT in occipital cortex is dependent on sensory experience during early development. The lack of visual experience must start influencing CT early on in development since, like adults, CB children have a thicker cortex than their sighted counterparts, while blind children with low vision do not (Inuggi et al., 2020; Ankeeta et al., 2021). Furthermore, these brain changes are long lasting, and not fully reversible, since people who had their sight restored by cataract surgery had decreased visual cortical area and higher CT than sighted individuals (Hölig et al., 2022). Indeed, a short and transient lack of visual experience early in life can have long lasting effects on brain development. Patients born with congenital cataracts exhibit long lasting changes in CT to the visual cortex and other visual areas, as well as resting state connectivity changes (Feng et al., 2021). Even slight changes to the quality of vision, such as in congenital achromatopsia, can also cause increases in CT in the visual cortex (Molz et al., 2022).

These first stages of development are characterized by delay (Begeer et al., 2014) or impairment (Gori et al., 2014) of certain abilities and altered brain development (Levtzion-Korach, 2001). During later stages of brain development, however, extensive training using touch and sound enables CB people to develop impressive skills that are linked to cross-modal activations of primary visual areas (Ptito et al., 2005; Merabet and Pascual-Leone, 2010; Voss et al., 2010; Kupers and Ptito, 2014). For example, CB possess enhanced verbal memory (Amedi et al., 2003), working memory (Heled et al., 2022), perceptual (Voss et al., 2004; Chebat et al., 2007; Arnaud et al., 2018), attention (Collignon et al., 2006), and cognitive (Fortin et al., 2008; Kupers et al., 2010; Chebat et al., 2015, 2017) skills compared to their sighted counterparts. This cross-modal recruitment keeps the primary visual cortex functional despite visual disuse for non-visual tasks (Amedi et al., 2003; Gougoux et al., 2005; Stevens et al., 2007; Jiang et al., 2009; Kupers and Ptito, 2014; Silva et al., 2018; Ptito et al., 2021). Variations in the thickness of the cortex in CB are linked to better performances on pitch and musical discrimination tasks. These changes are attributed to mechanisms of cross-modal training induced brain plasticity (Voss and Zatorre, 2012). Anurova et al. (2015) examined the relationship between CT and the magnitude of cortical activations in CB individuals during an attentionally demanding auditory task. They find that functional activations were negatively correlated with CT in all cortical areas involved in the auditory task (i.e., middle occipital gyrus, aSTG, pSTG). Meanwhile, cortical thinning in the CB brain is found in auditory and somatosensory areas, which is interpreted to reflect the interplay between developmental and adult training-induced plasticity (Park et al., 2009).

Reports of impaired performances and delayed brain development seem at odds with studies showing enhanced behavioral performances associated with cortical plastic changes in CB. In order to better understand the link between morphological changes and functional changes, it is imperative to look at several different measures of morphological changes. Gyrification (GY) is a complimentary measure to CT and is defined as the amount of cortical folding (Hogstrom et al., 2013). CT and GY have been shown to be complementary measures, the former being sensitive to environmental changes whereas the latter is assumed to reflect rather stable inherent morphological characteristics. Indeed, although GY also shows levels of increment and/or decrement during aging, these changes seem to be driven by loss of volume and surface area (White et al., 2010; Striedter et al., 2015). Furthermore, in terms of the link between structure and function, there are differential contributions of CT and GY to different forms of intelligence (Tadayon et al., 2020). It is possible that CT and GY may also be affected differently by complete absence of visual experience from birth. No study to date has investigated GY in CB adults, and this is also the first study to explore the link between CT and GY in CB. In sighted humans, CT is negatively related to GY (Gautam et al., 2015), and greater cortical GY is usually linked with enhanced cognitive abilities (Hogstrom et al., 2013; Gautam et al., 2015; Green et al., 2018). The mechanisms underlying changes in GY are still unknown (White et al., 2010), and the forces driving the relationship between GY and CT is still largely misunderstood (Striedter et al., 2015). There are many non-mutually exclusive hypotheses describing why the cortex folds during development (White et al., 2010; Striedter et al., 2015). The observation of increase or decrease in GY might lead to different hypotheses concerning the mechanisms underlying plastic changes of the cortex of the blind during development. For example, GY changes might underlie some of the functional-anatomical modulations observed in the visual areas of the blind following cross-modal associations (Amedi et al., 2004, 2010; Ptito et al., 2005). The functional-anatomical modulations observed in the visual areas of CB following cross-modal associations (Desikan et al., 2006; Luders et al., 2006; Dahnke et al., 2013) including CT might reflect GY changes, and possibly also changes in the nature of the relationship between CT and GY.

We report here for the first time GY changes in CB adults compared to sighted people, and the link between CT and GY. We examine the effects of congenital visual deprivation in cortical structures GY and CT by comparing a group of healthy CB individuals with age-matched sighted controls (SC). We hypothesize that the relationship between CT and GY in targeted areas should be consistent with the effects of early visual deprivation and also to cross-modal experience dependent plasticity.

## Materials and methods

### Participant

We studied 11 CB (6 males) and 16 SC (10 males). Blindness was of peripheral causes in all cases and without any cognitive or neurological comorbidities. CB participants were born blind and had no history of light perception, except GA who lost his sight at the age of 1. Average ages of CB and SC were 34 (range 20–48) and 30 (range 20–40) years. Demographic data and the causes of blindness are summarized in Table 1. All subjects gave informed consent and the study protocol was approved by the local research ethics committee.

**TABLE 1:** Demographic information of the congenitally blind group.

	Sex	Age	Causes of blindness
AK	M	30	Congenital–cause unknown.
DK	M	33	Congenital–cause unknown.
EM	F	33	Congenital–cause unknown.
EN	F	30	Congenital–cause unknown.
GA	F	32	Early (1 year)–Left eye didn’t develop during pregnancy, lost right eye when fell at the age of 1.
IG	M	42	Congenital–cause: Leiber disease.
MM	M	48	Congenital–cause: excess of oxygen.
OB	F	38	Congenital–cause: excess of oxygen.
OG	F	38	Congenital–cause: Rubella.
SH	M	30	Congenital–cause unknown.
SS	M	20	Congenital–cause unknown.

### Magnetic resonance imaging data acquisition

High resolution three-dimensional anatomical volumes were collected using MP-RAGE T1-weighted sequence with a magnetic field 3T GE Signa scanner (GE Medical Systems, USA). Typical parameters were: Field of View (FOV) 23 cm (RL) × 23 cm (VD) × 17 cm (AP); Fold over- axis: RL, data matrix: 160 × 160 × 144 zero-filled to 256 in all directions (approx. 1 mm isovoxel native data), TR/TE = 3 ms/2300 ms, flip angle = 8°, resulting in 160 scans per subject.

### Surface based morphometry analysis

Surface based morphometry analyses were computed using the CAT 12 toolbox (Structural Brain Mapping group, Jena University Hospital, Jena, Germany) implemented in SPM12 (Statistical Parametric Mapping, Institute of Neurology, London, UK). All T1-weighted images were corrected for bias e field inhomogeneities, then segmented into gray matter (GM), white matter (WM), and cerebrospinal fluid (CSF) (Gautam et al., 2015) and spatially normalized. The segmentation process was further extended by accounting for partial volume effects (Ptito et al., 2005), applying adaptive maximum *a posteriori* estimates. After pre-processing and in addition to visual checks for artifacts all scans passed an automated quality check protocol. CT and GY indices are determined by the projection based thickness (PBT) method (Desikan et al., 2006). GY index refers to the estimated surface complexity in 3D, indirectly reflecting the amount of cortical folding and an increase in surface areas housing more gray matter. For both indices, scans were smoothed with a Gaussian kernel of 20 mm (FWHM). Automated ROI based analysis was performed for both GY and CT. ROIs were based on cortical parcelation using Desikan-Killiany Atlas (Pietrini et al., 2004).

### Statistical analysis

Statistical analyses were carried out using the approach implemented in the CAT toolbox for SPM12. All values entered in the analysis were Z-transformed based on the mean and STD of the groups. We first examined group differences in CT by conducting a whole-brain analysis using a two-sample *t*-test adjusting for age and sex (*p* < 0.05, FDR correction). Comparisons between CB and SC groups was performed using independent *t*-tests separately for CT and GY indices. In all analyses, sex and age were entered as covariates in order to remove variance related to these potentially confounding variables. CAT 12 allows the estimation of surface parameters by surface-based atlas maps (Amedi et al., 2010). The atlas-based ROI analysis applies the information contained in the design matrix in order to extract parcellation based ROI values. False Discovery Rate correction was used to determine significant areas (FDR at *p* < 0.05).

We used paired *t*-tests to examine differences within each group concerning GY and CT. Data for all the areas except otherwise specified (see Table 2), were normally distributed according to Shapiro–Wilk test for normality (significant *p* < 0.05 suggesting deviation from normality).

**TABLE 2:** Desikan-Killiany (DK) Atlas brain areas (*p* < 0.05, FDR corrected) showing group differences (CB > SC) in Gyrification (GY).

	*T*-value	*Z*-value	*P*-value
**Left hemisphere**			
1. Left pars opercularis	3.77593	3.29	0.03
2. Left middle temporal	2.75599	2.53	0.048
3. Left temporal pole	2.66305	2.46	0.048
4. Left rostral middle frontal	2.64442	2.44	0.048
**Right hemisphere**			
5. Superior frontal	3.39	3.39	0.045
6. Pericalcarine	2.98	2.71	0.048
7. Frontal pole	2.68	2.47	0.048
8. Rostral middle frontal	2.85	2.61	0.048
9. Inferior temporal	2.68	2.47	0.048
10. Lateral occipital	2.64	2.438	0.048

#### General linear model testing the interaction between group, gyrification and cortical thickness

We first examined the relationship between GY and CT in cortical areas showing significant increase in GY (*p* < 0.05, FDR correction). A two-step regression approach was used to examine the relationship between GY and CT in the targeted cortical areas. The first model aimed at examining whether there was an association between GY and CT as a function of group and ROI. Therefore, GY was taken as dependent variable, CT, group and ROIs were chosen as the independent variables while adjusting for age and sex. The second model served as a *post-hoc* analysis examining the association between GY and CT within each ROI as a function of group (see: 31, for a similar approach). Checks for normality assumption were performed by using adequacy of Q-Q plots and residuals (see Supplementary material: Q-Q plots standardized residuals).

## Results

### Analysis of cortical thickness

In order to examine group differences in CT, we first conducted a whole-brain using a two-sample *t*-test adjusting for age and sex (FDR corrected *p* = 0.05). This analysis did not reveal any significant difference for the contrast SC > CB or for the reversed contrast CB > SC. Considering the small sample size used in the present study, and the fact that previous studies reported thickening of the cortex in CB individuals, we further explored group differences by using a less stringent exploratory threshold (*p* = 0.001 and *p* = 0.005, uncorrected) for the CB > SC contrast. Our analyses revealed a blob in the right fusiform area, and two additional blobs at the left middle occipital cortex and in the right superior motor area. Results for the whole-brain analysis are reported in (see Supplementary material: Analysis of CT, Supplementary Figure 1).

We also examined group differences by means of an atlas-based ROI analysis using FDR at *p* < 0.05. There was no significant group difference under FDR corrected threshold. The uncorrected threshold (*p* < 0.05) revealed differences for SC > CB and CB > SC contrasts. The results of this analyses are presented in full in Supplementary material. Although not reaching multiple comparisons threshold, CB > SC contrast revealed differences in the left and right occipital cortex. Despite not reaching statistical significance under FDR correction, the pattern from both whole-brain and ROI-based analysis are consistent with previous findings showing thickening of the occipital cortex in CB relative to controls (Pan et al., 2007; Jiang et al., 2009; Park et al., 2009; Voss and Zatorre, 2012).

### Analysis of gyrification

**FIGURE 1:**
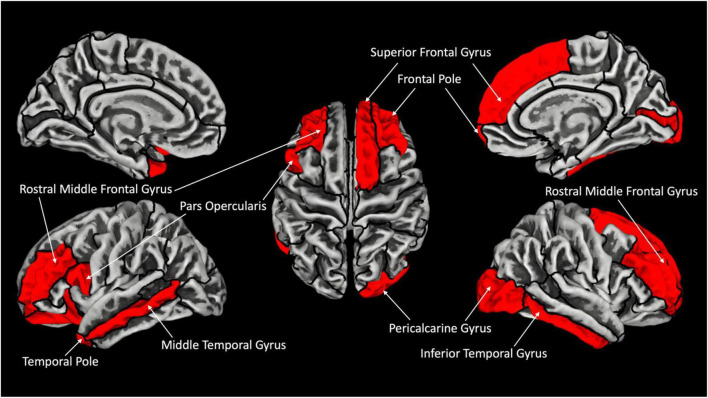
Increased gyrification in CB with respect to SC. Areas showing significant increase in gyrification are labeled in red (FDR corrected *p* < 0.05). Outline represent ROIs composing the Desikan–Kiliani Atlas (Desikan et al., 2006) (DK40) (Table 2 for full presentation of results).

In terms of group differences for GY (CB > SC) several brain areas are increased for the CB group (Figure 1). Namely, we find increases in the left hemisphere, the pars opercularis (BA44), temporal pole and middle temporal cortex, lateral orbitofrontal cortex and rostral middle frontal cortex. In the right hemisphere, we find a significant increase in GY in the following areas: superior frontal cortex, pericalcarine, frontal pole and inferior temporal, rostral frontal and lateral occipital cortex (Table 3 for the list of areas). The reverse contrast (SC > CB) did not reveal any differences.

**TABLE 3:** Pearson correlation coefficients between GY and CT for each DK atlas area showing increase in GY.

D.K Atlas label	Correlation between GY and CT	
	CB	SC
1. Left Pars opercularis (BA 44, 45, 47)	−0.53 (*p* = 0.09)	0.24 (*p* = 0.37)
2. Left middle temporal	−**0.64 (*p* = 0.03)**	−0.43 (*p* = 0.10)
3. Left temporal pole	−**0.81 (*p* = 0.002)**	−0.64 **(*p* < 0.007)**
4. Left rostral middle frontal	−0.15 (*p* = 0.70)	−0.32 (*p* = 0.23)
5. R superior frontal	**0.62 (*p* = 0.04)**	−0.11 (*p* = 0.70)
6. R pericalcarine	0.26 (*p* = 0.45)	−0.05 (*p* = 0.85)
7. R frontal pole	−0.67 (*p* = 0.02)*	−0.41 (*p* = 0.11)
8. R rostral middle frontal	−**0.85 (*p* = 0.009)**	−0.18 (*p* = 0.52)
9. R inferior temporal	−0.06 (*p* = 0.87)	−0.64 (*p* = 0.007)*
10. R lateral occipital	−0.27 (*p* = 0.42)	−0.16 (*p* = 0.57)

*Spearman correlation coefficient. Significant values are shown in bold font.

### Relationship between gyrification and cortical thickness in targeted cortical areas

We used Pearson’s correlation coefficient to determine which brain areas had significant correlations between GY and CT for the CB group. Most areas show a negative link between CT and GY except for the right superior frontal and pericalcarine areas for CB and the left pars opercularis for SC (Table 3). For SC, although the correlations follow a similar negative trend, it only reached significance in the left temporal pole and in the right inferior temporal cortex.

**FIGURE 2:**
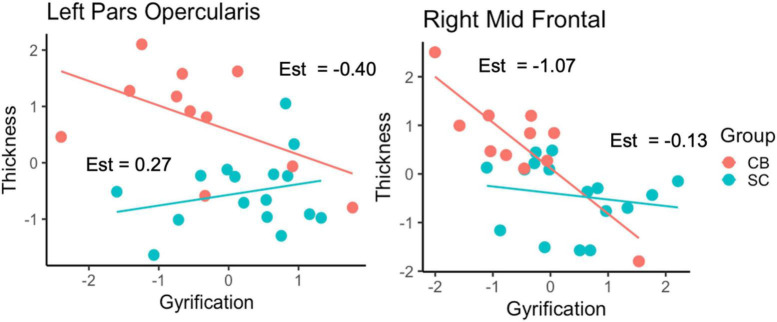
Relationship between GY and CT as a function of Group for the left pars opercularis and right rostral middle frontal cortex. Est = estimates of individual slopes.

We applied a linear regression analysis taking GY as dependent variable, Group, ROI and CT as regressors, while controlling for sex and age). We find a significant 3-way interaction *F*_(9,228)_ = 2.34, *p* = 0.015, as well as a significant main effect of CT, *F*_(1,228)_ = 23.52, *p* < 0.001. We further examined the association between GY and CT in a *post-hoc* analysis, for each ROI by means of separate linear regression models, taking GY as dependent variable, CT and group as independent variables adjusting for age and sex (Table 4). We observe a correlation between CT and GY changes for most areas except for the left rostral middle frontal, right pericalcarine, right inferior frontal, right inferior temporal and right calcarine areas. Of main relevance for the present report is the interaction involving Group and CT. We find that the level of CT in the left pars opercularis (BA 44) and in the right rostral middle frontal cortex had a significant impact on the amount of GY. We find a significant difference between the relationship between GY and CT between CB and SC groups. The impact of Group and CT as regressors of GY is illustrated in Figure 2.

**TABLE 4:** Results of linear regression models taking mean CT and GROUP as regressors, and the interaction term CT × Group adjusting for sex and age.

		Beta values	CI	*p*	Adjusted *R*^2^
L pars opercularis		–1.20	−1.83 to 0.56	0.001	0.48
	CT	–0.40	−0.96 to 0.16	0.001	
	**Group × CT**	**0.67**	**0**.**03 to 1.32**	**0.041**	
L middle temporal	Group	–0.68	−1.32 to 0.30	0.040	0.51
	CT	–0.93	−1.66 to −0.20	0.015	
	Group × CT	0.56	−0.23 to 1.35	0.157	
L temporal pole	Group	–0.44	−1.07 to 0.19	0.165	0.59
	CT	–0.93	−1.60 to 0.25	0.010	
	Group × CT	0.42	−0.31 to 1.16	0.244	
Left rostral middle frontal	Group	–0.84	−1.72 to 0.03	0.057	0.14
	CT	–0.13	−0.73 to 0.46	0.65	
	Group × CT	–0.26	−1.15 to −0.62	0.54	
R pericalcarine	Group	–1.21	−2.02 to −0.39	0.006	0.17
	CT	0.35	-0.32 to 1.03	0.29	
	Group × CT	–0.29	−1.10 to 0.52	0.46	
R frontal pole	Group	–0.52	−1.29 to 0.24	0.167	0.39
	CT	–0.75	−1.33 to −0.17	0.014	
	Group × CT	0.33	−0.38 to 1.05	0.345	
R rostral middle frontal	Group	–0.59	−1.23 to 0.06	0.074	
	CT	–1.07	−1.62 to −0.51	0.001	
	**Group × CT**	**0.94**	**0**.**26 to 1.61**	**0.009**	
R inferior temporal	Group	–0.76	−1.41 to 0.10	0.026	0.37
	CT	0.003	−0.51 to 0.51	0.991	
	Group × CT	–0.52	−1.15 to 0.12	0.104	
R lateral occipital	Group	–1.00	−1.73 to −0.28	0.009	0.45
	CT	–0.22	−0.69 to 0.24	0.33	
	Group × CT	–0.005	−0.64 to −0.63	0.988	

CI, confidence intervals. Beta values = unstandardized coefficients.

## Discussion

Although our results are limited by our small sample size and by the absence of any cognitive assessment data for our subjects (see “Limitations”), several distinct trends emerged from our data. We focus on three specific themes: comparisons of CT, cortical GY, and the relationship between CT and GY between our groups. We did not find statistically significant differences in CT between CB and SC in whole-brain analysis. However, using less stringent threshold, thickening of the occipital cortex was observed, consistent with previous findings (Pan et al., 2007; Jiang et al., 2009; Park et al., 2009; Voss and Zatorre, 2012). In terms of GY, several brain areas are increased for the CB group (Figure 1). We find that most of the areas that have increased GY are also correlated negatively with CT for the CB group (see Table 3). Of those areas where GY was increased, we find interactions involving Group and CT in the left pars opercularis (BA 44) and in the right rostral middle frontal cortex (Figure 2). We discuss our findings in the following sections in terms of the existing literature on the subject.

### Cortical thickness and gyrification following blindness in humans

Several brain areas are increased in terms of GY for the CB group (Figure 1). Namely, we find increases in the left the pars opercularis (BA44), temporal pole, middle temporal cortex, lateral orbitofrontal cortex, rostral middle frontal cortex, in the right superior frontal cortex, pericalcarine, frontal pole and inferior temporal, rostral frontal and lateral occipital cortex (Table 2). Interestingly, the temporal pole which we find increased in terms of GY, has been shown in previous reports to be reduced in terms of CT in CB (Park et al., 2009). Given that GY and CT are inversely related (Gautam et al., 2015), our results are congruent with these previous reports. Our report is congruent with CT increases in visual, sensory-motor, and auditory areas, and GI in bilateral visual cortex in CB children (Ankeeta et al., 2021).

The morphological changes we find in terms of GY and CT in the visual cortex of the CB are probably linked to mechanisms of general loss and compensatory plasticity. It has been shown that the lack of visual experience has an effect early on during development since CB children have increased CT compared to their sighted peers (Inuggi et al., 2020). Changes in CT have been described as “multi-systemic” since they affect several distinct brain areas. The areas that we find show increase GY in CB individuals are areas that have been consistently linked with cross modal activations in this population. For example, brain imaging studies in CB individuals show an increase in activation in these brain areas to non-visual stimuli (Amedi et al., 2003; Ptito et al., 2005; Kupers et al., 2010) recruiting the ventral stream for response to object recognition (Ptito et al., 2005, 2012; Striem-Amit et al., 2012), and the dorsal stream in responses to perceived motion of non-visual stimuli (Ricciardi et al., 2007; Ptito et al., 2009; Sani et al., 2010), and superior occipital cortex responses to spatial localization of sounds (Gougoux et al., 2005; Collignon et al., 2011). Our results show an increase in GY in the left pars opercularis, and rostral middle frontal cortex that carry language and memory for verbal stimuli. These areas are co-activated with the left triangular cortex and occipital cortex during a verbal-memory task in CB (Amedi et al., 2003), and connectivity between these areas and striate cortex is altered in congenital blindness (Butt et al., 2013), suggesting that mechanisms of cross-modal plasticity also influence measurements of GY in the CB brain.

### Relationship between gyrification and cortical thickness in targeted cortical areas

We find associations between GY and CT in various areas (Table 3). CT in the left pars opercularis (BA 44) and in the right rostral middle frontal cortex is correlated to the amount of GY in CB. Furthermore, the relationship between GY and CT is significantly different between CB and SC (Figure 2). These findings could suggest that GY and CT are not related in the same way for our two groups. We further explored the relationship between GY and CT by means of linear regression models, taking GY as dependent variable and CT and group as regressors (Table 4). We observe that CT is reliably correlated to GY changes in pars opercularis and in the right rostral middle frontal cortex. We further examined the association between GY and CT in a *post-hoc* analysis, for each ROI by means of separate linear regression models, taking GY as dependent variable, CT and group as independent variables (Table 4). Of main relevance for the present report is the interaction involving Group and CT. We find that the level of CT in the left pars opercularis (BA 44) and in the right rostral middle frontal cortex had a significant impact on the amount of GY (Figure 2).

We report a novel finding connecting two anatomical markers, GY and CT in CB individuals. Our findings are in line with previous work showing that there is increased thickness in cortical visual areas of the CB relative to SC including the pericalcarine sulcus, lingual gyrus, right rostral middle frontal gyrus, left caudate and anterior cingulate cortices (Park et al., 2009), and that both CB and LB have a thinner entorhinal cortex relative to SC (Jiang et al., 2009). The thickness of the cortex in CB is linked with better performance in a melody discrimination task (Voss and Zatorre, 2012). Changes in CT and GY are linked with duration of Braille education in CB and LB children (Ankeeta et al., 2021). These results were interpreted to reflect the interaction of cross-modal plasticity and mechanisms of synaptic pruning, possibly driving the relationship between CT and GY. We find that in these specific areas, GY changes are correlated to changes in CT.

The biological significance for the expansion of cortical surface, and the consequential increase in GY, is that it enables the increase in computational capacity of the brain (Toro and Burnod, 2005). Indeed, phylogenic increases in GY is associated with increased cognitive abilities and more complex behaviors (e.g., rodents versus primate cortex) (Gregory et al., 2016). Furthermore, cortical GY is associated with better cognitive function (Burgaleta et al., 2014; Gautam et al., 2015; Youn et al., 2021), and GY is inversely correlated with CT (McIntosh et al., 2009; Hogstrom et al., 2013; Gautam et al., 2015; Schmitt et al., 2022), meaning that in most brain areas increases in GY are usually, but not always accompanied by decreases in CT. CT and GY are both influenced by early experiences of maltreatment (Kelly et al., 2013), the acquisition of a new language (Vaughn et al., 2021) or being born preterm (Papini et al., 2020), showing its vast malleability. Different cognitive measures in older adults, revealed positive correlations between GY and cognitive abilities in the superior temporal gyrus, the insular cortex and in the post-central gyrus (Lamballais et al., 2020), and GY decreases with age (Frangou et al., 2022). The CB brain is greatly modified for processing tactile and auditory stimuli as well as braille reading, speech processing and verbal memory (Kujala et al., 1995; Röder et al., 2002; Amedi et al., 2003; Ptito et al., 2005). We show that the areas responsible for these processes also show differences in terms of GY and the relationship between CT and GY may also be an important measure of synaptic plasticity.

### Limiting factors

The present study is subject to various limitations inherent to working with small study populations. Different from previous studies, we did not find significant difference in CT in visual areas between CB and SC. We believe this null result could be possibly due to sample size issues since we did find differences in CT under less stringent threshold. Studies on blind subjects often deal with recruitment challenges and/or limited sample sizes. Moreover, included blind participants can be very heterogeneous in terms of blindness onset, duration, and cause, factors that may all affect functional outcomes. CB individuals represent an exceptionally rare population, even more so when strict selection requirements are enforced. Our sample of 12 participants can be considered as large and within the range of other classical brain morphometry studies in this population (for review see: Kupers and Ptito, 2014). Regardless, this is a major limiting factor of our study, and the relatively small sample size of CB participants forces us to be very cautious in our conclusions. Another major limiting factor is the absence of subject’s cognitive information. Indeed, it would be interesting to correlate changes in GY and CT with measures of performance in different cognitive tasks. Future studies should explore behavioral correlates of perceptual training and CT/GY changes, training induced brain plasticity and training of abilities in CB people and its impact on the relationship between GY and CT in a larger sample of participants including late blind and low vision participants.

## Conclusion

In this report, we show for the first time an increase in GY in several brain areas of CB adults compared to SC, and a negative relationship between GY and CT in the CB brain in several different cortical areas. We find that GY and CT covary differently in targeted areas, suggesting CB and SC may use these measures may be affected differently by the lack of visual experience, possibly reflecting disuse related mechanisms for CT changes, and training induced brain plasticity changes for GY changes in the CB. Furthermore, this differential relationship is highlighted by the fact that the relationship between GY and CT is not the same way in these two groups. Our results show that areas that are consistently implicated in cross-modal associations in CB are correlated in terms of CT/GY changes. Further exploration of ratio changes between GY and CT linked with perceptual learning skills in CB would enable us to disentangle the relation between cortical thickness, GY and behavioral abilities and the contributions of training induced plasticity vs. disuse related mechanisms.

## Data availability statement

The data supporting the conclusions of this article will be made available upon request by the corresponding author.

## Ethics statement

The studies involving human participants were reviewed and approved by The Hebrew University’s Ethics Committee. The patients/participants provided their written informed consent to participate in this study.

## Author contributions

IA, AA, and D-RC conceived and designed the experiments. IA, OY, KY, D-RC, and AA performed the experiments. IA, KY, D-RC, and AA analyzed the data. IA, KY, and D-RC wrote the manuscript. All authors contributed to the article and approved the submitted version.
