# Supplementary material for: Gyrification in relation to cortical thickness in the congenitally blind
Source: Front Neurosci. 2022 Nov 9;16:970878. doi: 10.3389/fnins.2022.970878 (PMC9682146; doi:10.3389/fnins.2022.970878)

**Supplementary Materials**

Cortical Thickness (CT) analysis:

**Figure 1.** Group differences in CT are displayed in Panel A (contrast SC > CB) and in Panel B (contrast CB > SC) using whole-brain analysis with p = 0.001, exploratory threshold. The contrast SC > CB, revealed a blob at right inferior operculum [60,9,12], T = 4.84, Z = 3.98, uncorrected p = 0.001. The reversed contrast CB > SC revealed a blob at right fusiform area [27, -79,-16], T = 4.21, Z = 3.59, uncorrected p = 0.001. Because of our small sample size, we further explored CT on the congenitally blind (CB > SC) taking a less stringent p value (p =.005, Panel C). two additional areas emerged, one at Left middle occipital cortex [-17, -102,5], T = 3.44, Z = 3.06, p = 0.001, and another in the right superior motor area [4,1,64], T = 3.36, Z = 3.00, p = 0.001.

**A B**

**
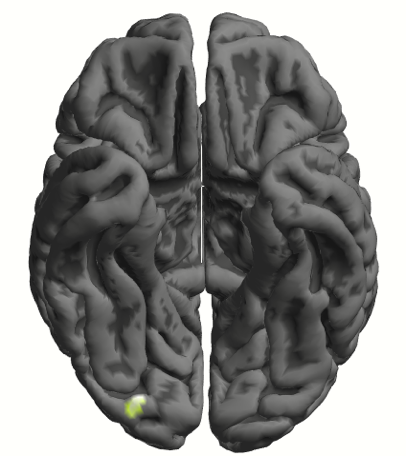

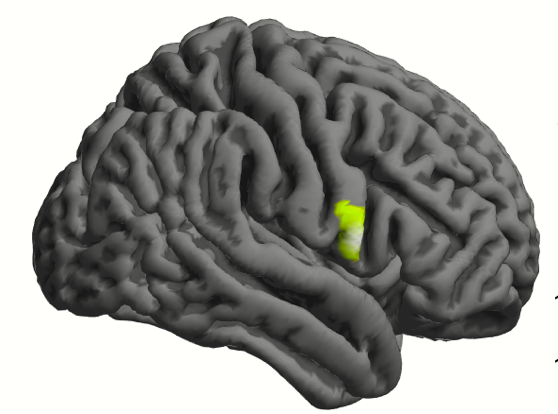
**

**C**


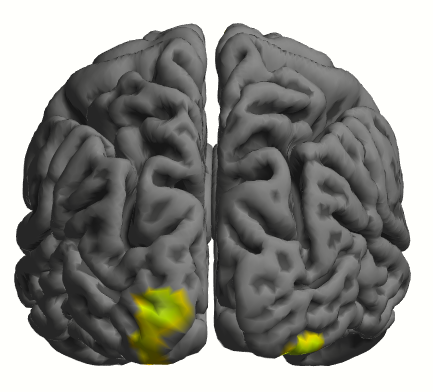

Supplement: Supplementary file 1 [file Table_1.DOCX]
